# Supplementary material for: Phenotypic bistability in Escherichia coli's central carbon metabolism
Source: Mol Syst Biol. 2014 Jul 1;10(7):736. doi: 10.15252/msb.20135022 (PMC4299493; doi:10.15252/msb.20135022)
Supplement: Supplementary file 1 — Supplementary Figure S1 [file msb0010-0736-sd1.pdf]

Supplementary Figure S1: Responsive diversification occurs for various gluconeogenic carbon sources

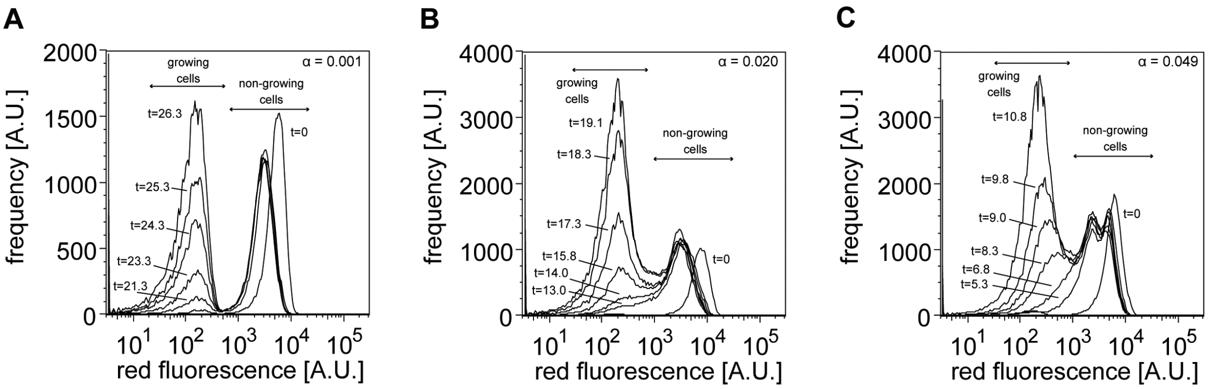

(A-C) Fluoresence intensity distributions at multiple time points after the shift from glucose to 2 g L^{-1} fumarate (A), succinate (B) and malate (C). α values (fractions of growing cells) were determined by a model-based approach (cf. Supplementary Materials and Methods). The results reveal that two phenotypic subpopulations occur following shifts to each of these gluconeogenic substrates. Therefore, responsive diversification is of a general nature, occurring upon substrate shifts from glucose to various gluconeogenic substrates.
