# Supplementary material for: Phenotypic bistability in Escherichia coli's central carbon metabolism
Source: Mol Syst Biol. 2014 Jul 1;10(7):736. doi: 10.15252/msb.20135022 (PMC4299493; doi:10.15252/msb.20135022)
Supplement: Supplementary file 2 — Supplementary Figure S2 [file msb0010-0736-sd2.pdf]

Supplementary Figure S2: Validation of cell-staining assay

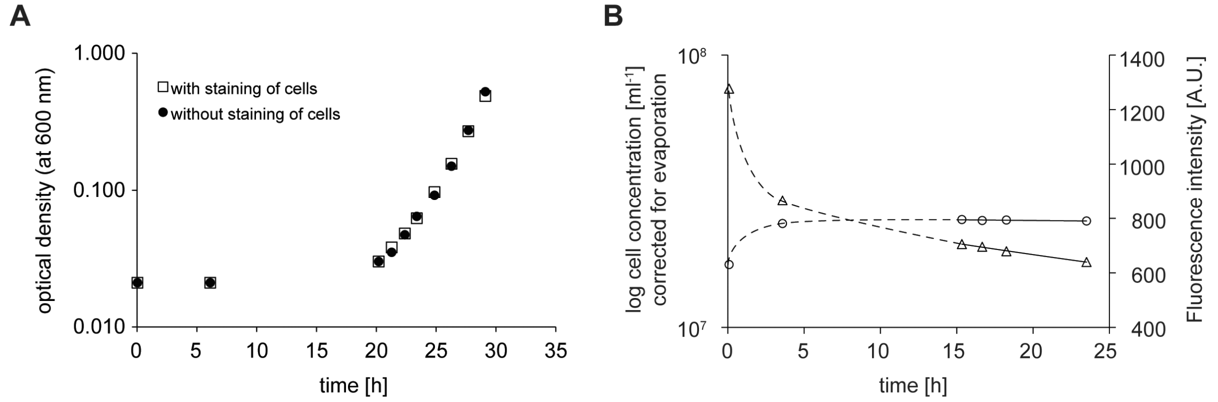

(A) Exclusion of toxic effects of the staining procedure on cell viability. The growth curves of stained and unstained cells following a shift from glucose to 2 g L^{-1} fumarate are identical. Thus, if the non-growing cells of the stained population were an artifact of the membrane staining (e.g. would be killed through the staining), then the overall cell growth dynamics would be different, i.e. the stained population's growth curve would exhibit a longer lag time. But this is not the case: this result demonstrates that the membrane staining does not affect the population's response to the substrate shift.

(B) Determination of unspecific dye loss. Progression of the cell concentration (circles) and fluoresence intensity of the cell population (triangles) over time in a medium without carbon source. To check if fluoresent dye attached to the cells is lost to the surrounding media over time, bleached or if its intensity is generally decreased in any other way, glucose grown cells were stained and transferred to M9 medium without carbon source. As expected from the fact that there was no carbon source present in the second culture, the cell concentration remained constant (after an initial period of reductive cell division (Nystrom, 2004)). After completion of the reductive cell division, the fluoresence intensity of the cells decreased with a constant rate of 0.015 h^{-1}. We attribute this intensity decrease to unspecific dye loss and deem it marginal compared to the growth rate.

Nystrom, T. (2004). Stationary-phase physiology. Annu. Rev. Microbiol. *58,* 161-181.
