# Supplementary material for: Phenotypic bistability in Escherichia coli's central carbon metabolism
Source: Mol Syst Biol. 2014 Jul 1;10(7):736. doi: 10.15252/msb.20135022 (PMC4299493; doi:10.15252/msb.20135022)
Supplement: Supplementary file 3 — Supplementary Figure S3 [file msb0010-0736-sd3.pdf]

Supplementary Figure S3: Non-growing cells are viable cells and can regrow on glucose

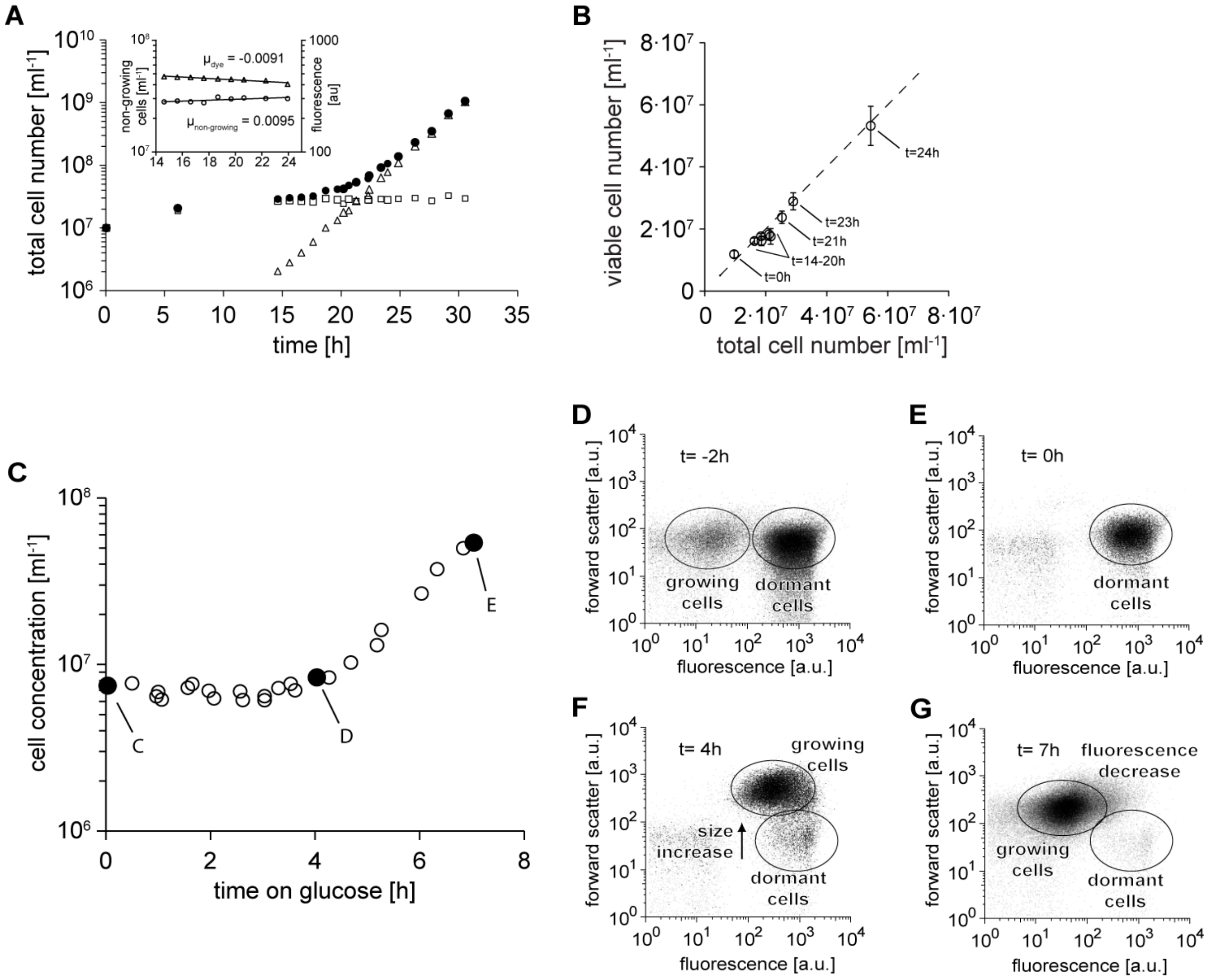

(A) Cell counts of non-growing (empty squares), growing (empty triangles) and the total number of cells (full circles) after an isogenic cell population was shifted from glucose to 2 g L^{-1} fumarate.

(B) Total cell counts by flow cytometry plotted against viable cell counts by dilution plate assay after a glucose-to-fumarate shift after 0, 2, and in 2 hour intervals between 14 and 26 hours after the shift (dashed line: diagonal). These measurements were performed in triplicates.

(C) Growth curve of dormant cells on glucose minimal medium. These dormant cells were obtained 18 h after a glucose-to-fumarate shift (in which the cells were stained with the fluorescent dye) and a 2h treatment with ampicillin to lyse the growing cells. This time period lasted from t=-2h to t=0. Dormant cells were re-suspended in glucose minimal medium at t=0.

(D-G): Dot plots scaled to show the same number of events. D: Cells 18 hours after a shift from glucose to fumarate displaying the typical growing and dormant phenotypes (t=-2h). E: Cells after 2 hours of ampicillin exposure (from t=-2h to t=0). E/F: Dormant cells re-growing in glucose minimal medium. After a lag phase of about 4 hours, during which the cells increased in size, the cells began to divide and showed the typical decrease of membrane dye intensity, while their number increased. Counting of the cells that remain non-growing on glucose showed that most of the cells (75%±7; resumed growth on glucose. We think that the reason why not all cells resumed growth might be attributed to the fact that still some non-growing cells were being killed by ampicillin. Evidence comes from the observation that the here found number of 75% is comparable to the number we found in the experiment shown in Supplementary Figure S10B.
