# Supplementary material for: Phenotypic bistability in Escherichia coli's central carbon metabolism
Source: Mol Syst Biol. 2014 Jul 1;10(7):736. doi: 10.15252/msb.20135022 (PMC4299493; doi:10.15252/msb.20135022)
Supplement: Supplementary file 4 — Supplementary Figure S4 [file msb0010-0736-sd4.pdf]

Supplementary Figure S4: Exclusion of stochastic switching

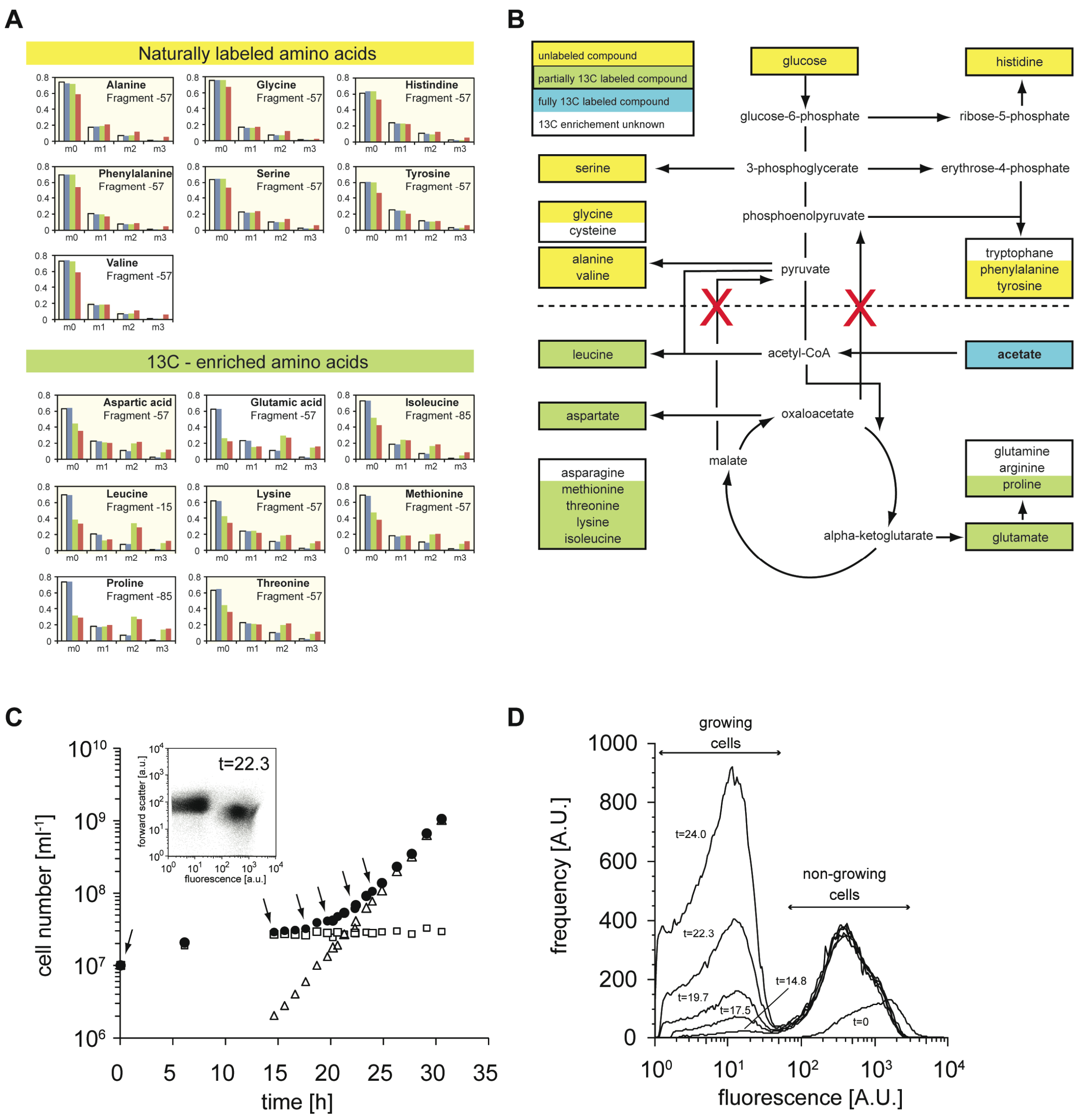

(A) Mass distribution vectors (MDV's) of amino acids. The index $i = 0,1,2,3$ of a MDV indicates the number of the isotope's ^{13}C atoms. White columns: theoretical values of the natural labeling pattern. Blue columns: negative controls. Green columns: actual samples. Red columns: positive controls. Seven amino acids exhibited a natural (i.e. no) labeling pattern - their MDV's were close to the theoretical values and negative controls. Eight amino acids exhibited a ^{13}C-enriched labeling pattern - their MDV's were close to the positive control.

(B) ^{13}C-enriched amino acids were mapped to their respective precursor metabolites in central carbon metabolism. Yellow boxes: naturally labeled compounds. Green boxes: ^{13}C-enriched compounds. Blue box: fully ^{13}C-labeled compound. White boxes, compounds with unknown isotopomer distribution. A ^{13}C -enrichment occurs only in those amino acids (green boxes) that are derived from metabolites below pyruvate. A natural labeling pattern is exhibited by those amino acids (yellow boxes) that are derived from metabolites above pyruvate.

(C-D) No inter-phenotype switching between growing and non-growing cells occured after the initial decision at the time of the carbon source shift. (C) Growth curve of glucose adapted and stained cells shifted to 2 g L^{-1} fumarate minimal medium. Growing and non-growing cells were discerned based on their staining intensity. Full circles: total cell number, empty squares: non-growing cells, empty triangles: growing cells. (D) Histograms of the fluorescence distribution of the cell population at different time points. Two observations demonstrate that the chosen phenotypes were stable after the carbon source shift: (i) The number of non-growing cells remained constant during the growth of the culture. If non-growing cells were to resume growth, the decrease in their fluorescence would lead to a decrease in the number of cells with high red fluorescence. (ii) The fluorescence histogram of the culture shows bimodal distribution with a clear gap between the two populations. If growing cells stopped to grow, there would be no such gap as it would be filled up with cells having switched to the non-growing phenotype at intermediate fluorescence levels. Equally, if non-growing cells would stochastically start switching to the growing phenotype, we would see them filling the gap between the two populations.
