# Supplementary material for: Phenotypic bistability in Escherichia coli's central carbon metabolism
Source: Mol Syst Biol. 2014 Jul 1;10(7):736. doi: 10.15252/msb.20135022 (PMC4299493; doi:10.15252/msb.20135022)
Supplement: Supplementary file 5 — Supplementary Figure S5 [file msb0010-0736-sd5.pdf]

Supplementary Figure S5: No pre-conditioning of the cell population for acetate consumption

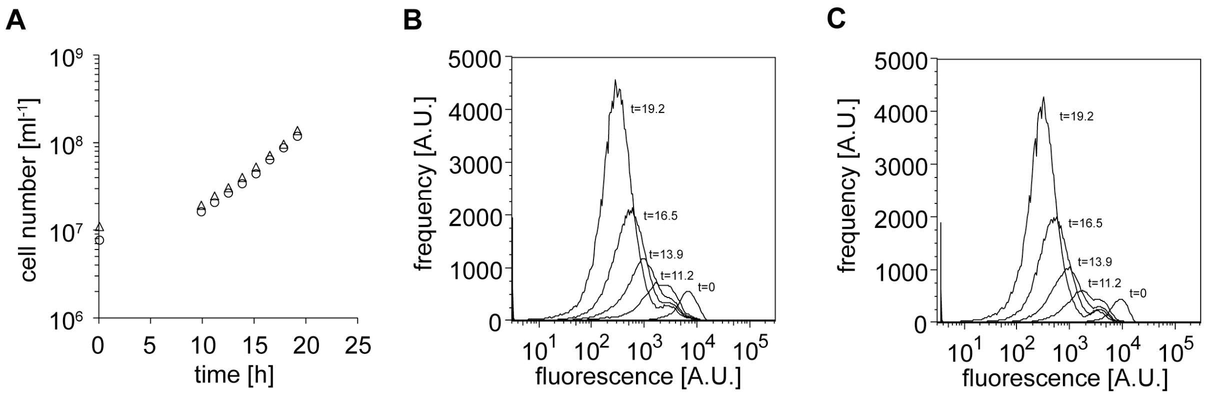

(A) Cell counts in the 0.75 g L^{-1} acetate culture (triangles: cells coming from normal glucose medium; circles: cell coming from glucose medium with 0.5 g L^{-1} acetate added).

(B) Fluorescence intensity distributions of cells on acetate, with cells derived from the normal glucose culture.

(C) Fluorescence intensity distributions of cells on acetate, with cells derived from the glucose culture with 0.5 g L^{-1} acetate added. The fact that these curves are absolutely identical demonstrates that the addition of acetate to the glucose culture did not change the population behavior and therefore did not pre-condition (prime) the cells for the later (re)-consumption of acetate.
