# Supplementary material for: Phenotypic bistability in Escherichia coli's central carbon metabolism
Source: Mol Syst Biol. 2014 Jul 1;10(7):736. doi: 10.15252/msb.20135022 (PMC4299493; doi:10.15252/msb.20135022)
Supplement: Supplementary file 6 — Supplementary Figure S6 [file msb0010-0736-sd6.pdf]

Supplementary Figure S6: Fumarate uptake rates at different induction levels of the fumarate transporter DctA

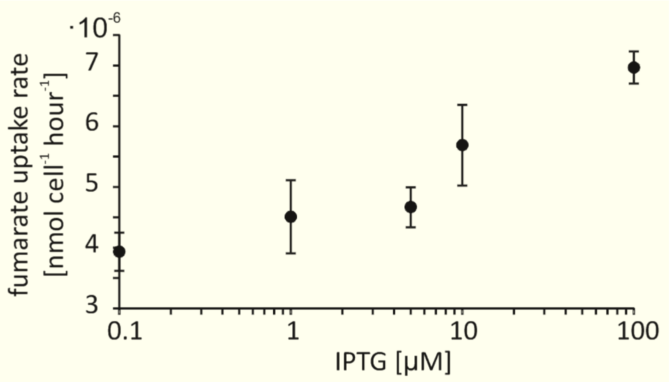

Steady-state fumarate uptake rates at 2 g L^{-1} fumarate as a function of IPTG concentrations in a DctA deletion strain carrying plasmid pP_{tac}-dctA demonstrating that the fumarate uptake rate can be modulated by inducing expression of the DtcA fumarate transporter. Experiments were performed in triplicates and error bars indicate one standard deviation.
