# Supplementary material for: Phenotypic bistability in Escherichia coli's central carbon metabolism
Source: Mol Syst Biol. 2014 Jul 1;10(7):736. doi: 10.15252/msb.20135022 (PMC4299493; doi:10.15252/msb.20135022)
Supplement: Supplementary file 7 — Supplementary Figure S7 [file msb0010-0736-sd7.pdf]

Supplementary Figure S7: Lag phase dependance on alpha

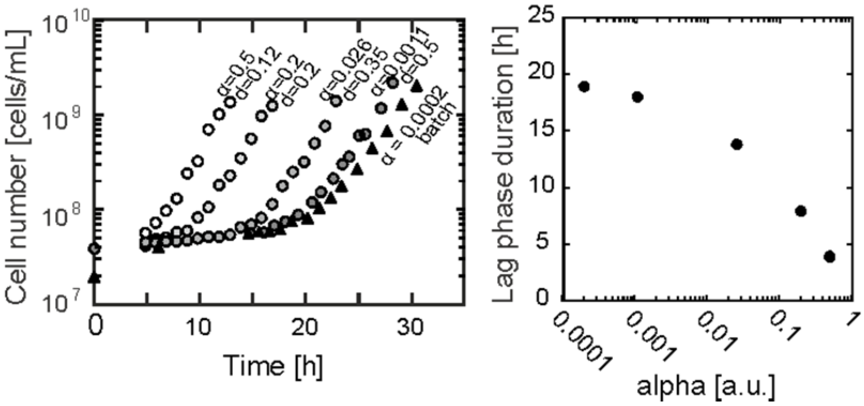

(A) Growth curves of cells grown in glucose-limited chemostats, stained and switched to 2 g L^{-1} fumarate; and (B) apparent lag phase duration dependence on alpha. Lag phase duration was determined by finding the time at which a function describing exponential phase of growth and the initial cell count (after reductive division, in case of the batch culture) have equal values. While alpha can explain most of the lag phase, some biochemical adaptation is still needed for the cells to start growth on the new carbon source.
