# Supplementary material for: Phenotypic bistability in Escherichia coli's central carbon metabolism
Source: Mol Syst Biol. 2014 Jul 1;10(7):736. doi: 10.15252/msb.20135022 (PMC4299493; doi:10.15252/msb.20135022)
Supplement: Supplementary file 8 — Supplementary Figure S8 [file msb0010-0736-sd8.pdf]

Supplementary Figure S8: Bifurcation analysis

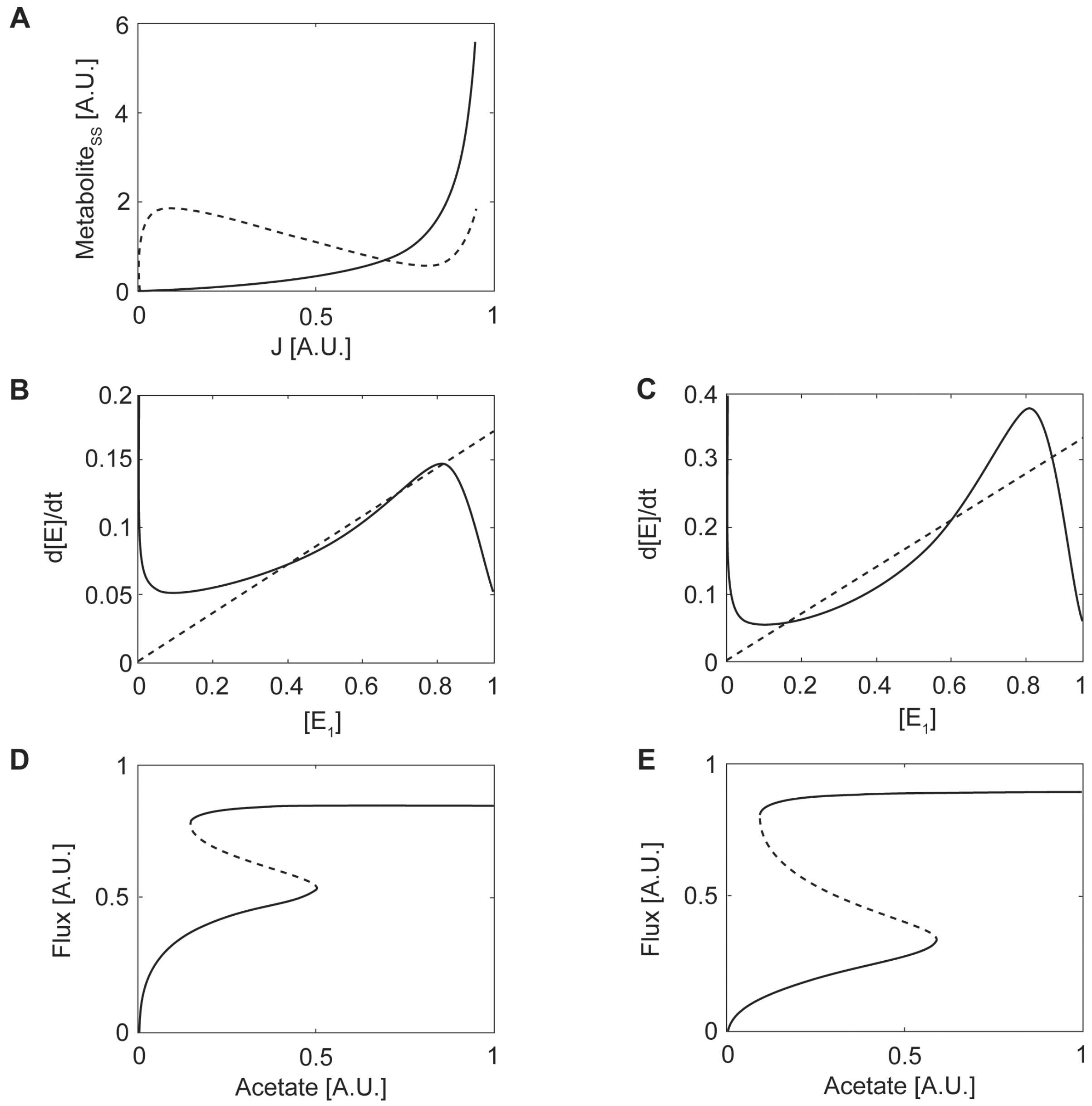

(A) Steady state *X* (solid line) and *FBP* (dotted line) metabolite concentrations over steady state flux, which are identical for both parabolic ($n_e = 1$) and sigmoidal ($n_e = 2$) influence of *FBP* on *E* production.

(B-C) *E* production (solid line) and degradation/dilution (dotted line) curves for parabolic (B) and sigmoidal regulation (C) of *E* production by *FBP*. These two figures were generated assuming saturation of the enzyme *E* with its substrate acetate (*acetate* >>$K_{E,acetate}$), such that $v_E = k_{E,cat} \cdot E$.

(D-E) Bifurcation diagrams showing stable (solid lines) and unstable (dotted lines) steady states for parabolic (D) and sigmoidal regulation (E) of *E* production by *FBP*.
