# Supplementary material for: Phenotypic bistability in Escherichia coli's central carbon metabolism
Source: Mol Syst Biol. 2014 Jul 1;10(7):736. doi: 10.15252/msb.20135022 (PMC4299493; doi:10.15252/msb.20135022)
Supplement: Supplementary file 9 — Supplementary Figure S9 [file msb0010-0736-sd9.pdf]

Supplementary Figure S9: Complementary switch experiments

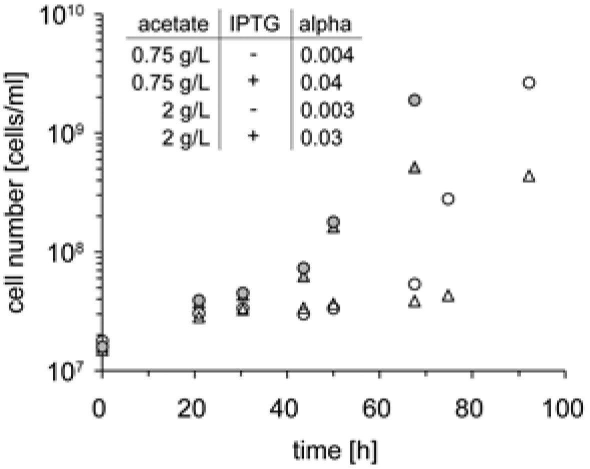

Overexpression of the alternative isoenzyme or parallel pathway can restore wild-type-like α values. If the drastically reduced α values are indeed due to the introduced gene expression bottlenecks, then wild-type-like α values should be restored through overexpression of the alternative isoenzyme or parallel pathway. *acnA* overexpression in an *acnB* mutant reduced the lag phase after a glucose to acetate shift. The growth curves of the *acnB* mutant containing the *acnA* overexpression plasmid are plotted together with the determined alpha values (circles: 2 g L^{-1} acetate, triangles: 0.75 g L^{-1} acetate, filled symbols: +0.1mM IPTG, empty symbols: no IPTG). The behavior of the Δ*ppsA* was fully reverted to wild-type behavior through overexpression of *pckA* (Kao et al., 2005) and the extensive apparent 'lag times' of Δ*maeBsfcA* were markedly reduced through overexpression of *ppsA* (Kao et al., 2005).

Kao, K.C., Tran, L.M., and Liao, J.C. (2005). A global regulatory role of gluconeogenic genes in Escherichia coli revealed by transcriptome network analysis. J. Biol. Chem. *280,* 36079-36087.
