# Supplementary material for: Phenotypic bistability in Escherichia coli's central carbon metabolism
Source: Mol Syst Biol. 2014 Jul 1;10(7):736. doi: 10.15252/msb.20135022 (PMC4299493; doi:10.15252/msb.20135022)
Supplement: Supplementary file 10 — Supplementary Figure S10 [file msb0010-0736-sd10.pdf]

Supplementary Figure S10: Non-growing cells are dormant persister cells

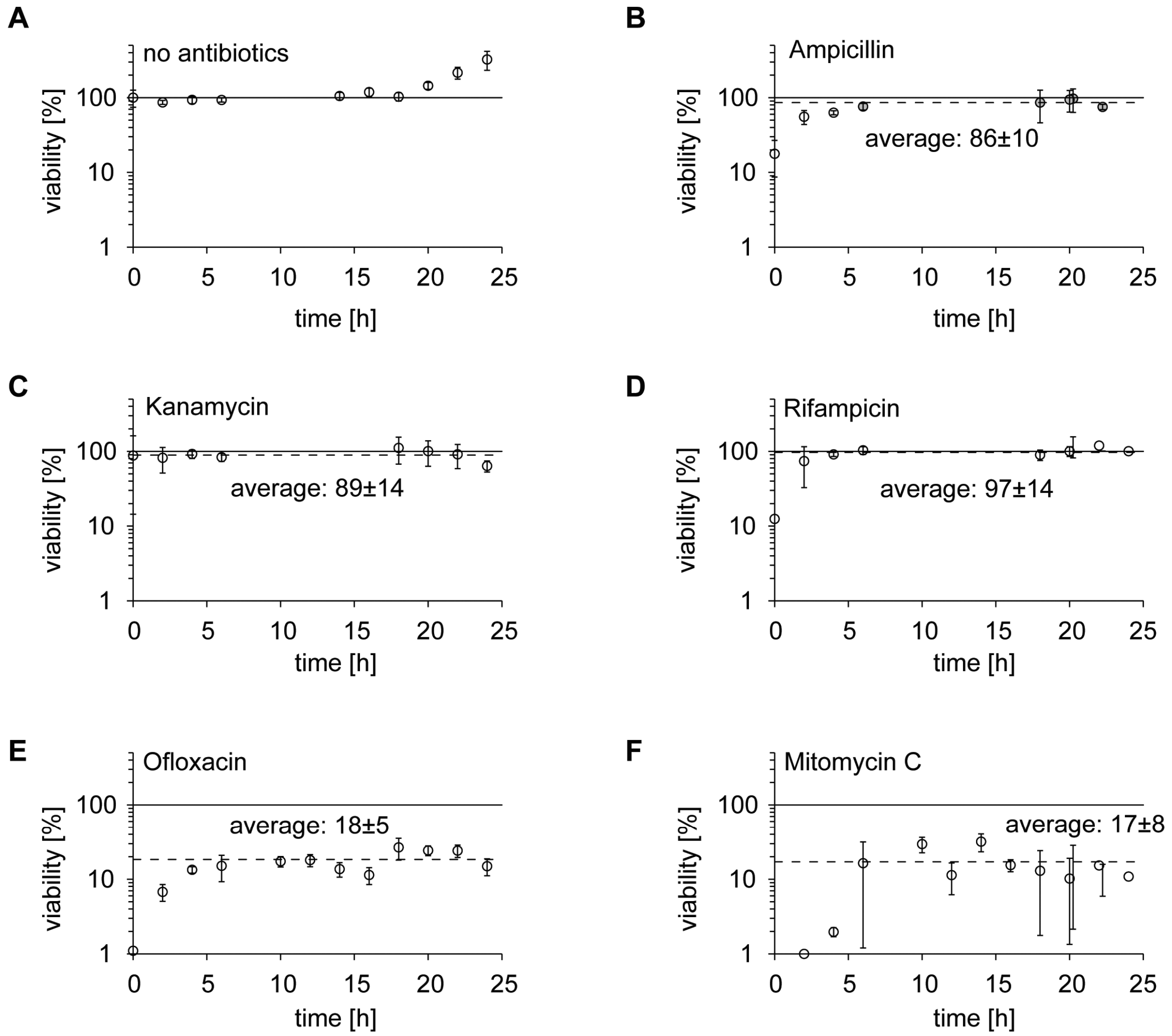

(A-F) Development of antibiotic resistance in dormant cells. After a glucose-to-fumarate (2 g L^{-1}) shift and subsequent exposure to different antibiotics the percentage of viable cells relative to all cells is plotted over time. The average of the total cell number after a glucose-to-fumarate shift from 2 to 18 hours is set as 100%. As during this period almost all cells were non-growing (cf. Supplementary Fig. S3A), the here indicated viability can be considered the survival percentage of the non-growing cells. Antibiotic survival was determined by 2 hours exposure to the indicated antibiotics (ampicillin 50 µg/mL, ofloxacin 5 µg/mL, kanamycin 25 µg/mL, mitomycin C 5 µg/mL, rifampicin 50 µg/mL) and subsequent dilution plating on cell counting plates, performed at least in triplicate.
