# Supplementary material for: Phenotypic bistability in Escherichia coli's central carbon metabolism
Source: Mol Syst Biol. 2014 Jul 1;10(7):736. doi: 10.15252/msb.20135022 (PMC4299493; doi:10.15252/msb.20135022)
Supplement: Supplementary file 11 — Supplementary Table S1 [file msb0010-0736-sd11.pdf]

Supplementary Table S1: Population fractions α occuring at various other carbon source switches

Strain	First substrate	Second substrate	α
Wild type	acetate, 2 g L^{-1}	glucose, 2 g L^{-1}	0.95*
	acetate, 1.5 g L^{-1} †	acetate, 1.5 g L^{-1}	0.99*
	glucose-6-phosphate, 5 g L^{-1}	alanine, 2 g L^{-1}	0.42
	N-acetyl-D-glucosoamine, 5 g L^{-1}	ribose, 2 g L^{-1}	0.36
	lactate, 2 g L^{-1}	fumarate, 2 g L^{-1}	0.015
	pyruvate, 2 g L^{-1}	fumarate, 2 g L^{-1}	0.008
ΔmaeB sfcA	glucose, 5 g L^{-1}	acetate, 2 g L^{-1} (1st round‡)	0.085
	glucose, 5 g L^{-1}	acetate, 2 g L^{-1} (2nd round‡)	0.065

*While for most experiments the procedure mentioned in the Supplementary Materials and Methods (‘Model-based experimental determination of fraction of adapting cells’) was used to estimate the α value, this was not possible in those cases, where all or almost all cells started to grow on the second carbon source, because in these experiment basically only one Gaussian distribution of fluorescence intensities is visible in the flow cytometric data. Thus, in the cases indicated with an asterisk, α values were estimated using the equation $\alpha = \frac{X_g(t=x) / 2^{div}}{X_t(t=0)}$, with $X_g(t=x)$ being the number of growing cells at a time point $t = x$ when basically only growing cells are present, $X_t(t=0)$ being the total number of cells right after the switch, and div the number of divisions that the growing cells made after the switch as estimated from the loss in fluoresence considering the cells background fluoresence intensity. Note that α values close to 1 are difficult to determine and these values could in fact also be 1.

†Acetate concentration at the time point of the switch.

‡In order to exclude mutations as a cause for the phenotypic subpopulations with the *maeBsfcA* mutant cells from a single-colony from an LB-agar plate were grown overnight on glucose to mid-exponential

phase (OD~0.5), then stained and shifted to acetate 2 g L^{-1}. In late exponential phase of the acetate culture (OD ~0.8, after ~35 hours), where more than 95% of the cells exhibit the growing phenotype, cultures were diluted 100-fold to fresh medium with acetate 2 g L^{-1}. The new culture was then incubated until OD~0.8, where ultimately more than 99.95% of cells exhibit the growing phenotype. Cells from that culture were streaked out on a LB-agar plate. A single colony from this plate was used for a second round of the carbon source shift experiment, following the same protocol. Identical growth rates of the growing populations and identical α values within the usual measurement uncertainty range were found in the consecutive rounds, thus excluding mutations as the cause for the observation. For the *ppsA* mutant, a suppressor mutation was already ruled out to be the cause for the extended lag phase (Kao et al., 2005).

Kao, K.C., Tran, L.M., and Liao, J.C. (2005). A global regulatory role of gluconeogenic genes in Escherichia coli revealed by transcriptome network analysis. J. Biol. Chem. *280,* 36079-36087.
