# Supplementary material for: Phenotypic bistability in Escherichia coli's central carbon metabolism
Source: Mol Syst Biol. 2014 Jul 1;10(7):736. doi: 10.15252/msb.20135022 (PMC4299493; doi:10.15252/msb.20135022)
Supplement: Supplementary file 12 — Supplementary Table S2 [file msb0010-0736-sd12.pdf]

Supplementary Table s2: Phenotypes of transcription factor deletion mutants on glucose and acetate

**Strain / conditions**	**Phenotype on glucose**	**Phenotype on acetate**
Δ*cra*	growth rate as wild-type	no growth
Δ*cra* + 0.5 mmol/L cAMP	growth rate as wild-type	no growth
Δ*cra* + 1 mmol/L cAMP	growth rate as wild-type	no growth
Δ*cra* + 5 mmol/L cAMP	growth rate as wild-type	no growth
Δ*crp*	slower growth rate	slower growth rate
Δ*arcA*	growth rate as wild-type	growth rate as wild-type
Δ*iclR*	growth rate as wild-type	growth rate as wild-type

To test whether the transcription factor Crp could rescue the Cra deletion mutant when switching to acetate, we externally induced Crp activity by adding cAMP at the indicated concentrations to the glucose medium before the shift and to the acetate medium after the shift. The used concentrations were earlier shown to fully activate Crp (Sasson et al., 2012). We monitored cell behavior on acetate for over 80 hours. As the growth phenotype of the *cra* deletion mutant on acetate could not be rescued through the addition of cAMP (and thus through increased Crp activity), it can be excluded that Crp has a major role in generating the bistable phenotypes.

Sasson, V., Shachrai, I., Bren, A., Dekel, E., and Alon, U. (2012). Mode of regulation and the insulation of bacterial gene expression. Mol. Cell *46,* 399-407.
