# Supplementary material for: Phenotypic bistability in Escherichia coli's central carbon metabolism
Source: Mol Syst Biol. 2014 Jul 1;10(7):736. doi: 10.15252/msb.20135022 (PMC4299493; doi:10.15252/msb.20135022)
Supplement: Supplementary file 13 — Supplementary Materials and Methods [file msb0010-0736-sd13.pdf]

Supplementary Materials and Methods

Rationale and control of the single cell staining experiment

In this work, we introduced and used a novel experimental method to uncover different growth phenotypes (main text). The method involves staining of *E. coli* cells with a fluorescent membrane-intercalating dye (cf. Materials and Methods), following the cells' fluoresence intensity distribution over time with flow cytometry (cf. Materials and Methods) and analyzing the data with a mathematical model (cf. Supplementary Materials and Methods). With this method, we can determine the growth rate of the growing population as well as the fraction of the cells adapting to the new carbon source, α (cf. Supplementary Materials and Methods).

The underlying idea of the cell staining experiment is the following: A cell that grows and divides distributes the dye molecules attached to the cell membrane equally between the two daugther cells. The rate of dye loss (after subtraction of the unspecific dye loss) can then be used as proxy for the cells' growth rate. We performed an experiment that shows that this underlying idea is correct, i.e. that the growth rate correlates with the rate of fluorescence intensity loss: Cells were grown in M9-glucose medium to exponential phase, then stained (cf. Materials and Methods) and re-inoculated in fresh M9-glucose medium. Analysis of the fluorescence intensity distribution of the cells at different time points (determined by flow cytometric analyses) revealed that the rate of intensity decrease after subtraction of unspecific dye loss (cf. Supplementary Figure S2B) corresponds to the growth rate of the cells. This also means that if the initial staining intensity is known, the amount of divisions a cell has underwent can be determined from its staining intensity at a certain later time point (as long as the fluorescence intensity is still above the background fluoresence of the cell).

Model-based experimental determination of fraction of adapting cells

The following section describes how we determined the fractions of the cells adapting to gluconeogenic growth, α, and their growth rates μ_g. In essence, we determined these values by fitting a bi-Gaussian model (with α and μ_g as parameters) to the measured time progressions of the total populations' fluorescence intensity distributions obtained after staining the cells with the fluoresent dye. A representation of such a fit is shown in Fig. 2B of the main text.

Specifically, the determination of α and μ_g requires (i) the cell count concentration $c(t_0)$ and the fluorescence intensity distribution of the total population $\Delta(t_0)$ measured immediately after the substrate shift at $t = t_0$, and (ii) a sequence of k cell count concentrations and fluorescence intensity distributions measured at time instants $t = t_i$, $i = 1 \ldots k$ within the observation window $[t_1, t_k]$, designated $c(t_i)$ and $\Delta(t_i)$ with $i = 1 \ldots k$.

To obtain the $k+1$ cell count concentrations c and fluorescence intensity distributions Δ , the raw data provided by flow cytometry measurements is processed by

1. smoothing the fluorescence data with a 3-point moving average filter,
2. discarding the lowest 200 of in total 1024 fluorescence intensity bins due to unacceptably low signal-to-noise ratios,
3. correcting the fluorescence intensities for background noise by subtracting a noise distribution-approximating, decaying exponential from the total signal.
4. correcting the measured cell count concentrations with the culture's evaporation rate of 0.142 ml/h, and
5. scaling the smoothed and noise-corrected fluorescence intensity distributions with the evaporation-corrected cell count concentrations.

The measured, time-dependent, total population's fluorescence intensity distribution data $\Delta(t)$ is approximated with a model, $\mathrm{M}(t)$. In the model, the time-dependent, total population's fluorescence

intensity distributions are given as the sum of the two time-dependent distributions $\mathrm{M}_g(t)$ and $\mathrm{M}_n(t)$ of the 'growing' and 'non-growing' subpopulations, respectively. Hence,

$$\mathrm{M}(t) = \mathrm{M}_g(t) + \mathrm{M}_n(t) . \tag{1}$$

Both $\mathrm{M}_g(t)$ and $\mathrm{M}_n(t)$ are modeled as Gaussian distributions $\mathrm{N}(m,\sigma)$ with time-dependent mean intensities $m_g(t)$ and $m_n(t)$, respectively, and time-invariant standard deviations σ_g and σ_n, respectively. The areas of the Gaussians increase exponentially with the subpopulations' growth rates μ_g and μ_n, respectively. The areas of the $\mathrm{M}_g(t_1)$ and $\mathrm{M}_n(t_1)$-distributions are given by the cell counts $c_{g,1}$ and $c_{n,1}$, respectively. Hence, the time progressions of $\mathrm{M}_g(t)$ and $\mathrm{M}_n(t)$ within the observation window $[t_1, t_k]$ are given by

$$\begin{aligned} \mathrm{M}_g(t) &= c_{g,1}\, \mathrm{e}^{\mu_g (t-t_1)}\, \mathrm{N}(m_g(t),\sigma_g)[256\log_{10}(\cdot)] \\ \mathrm{M}_n(t) &= c_{n,1}\, \mathrm{e}^{\mu_n (t-t_1)}\, \mathrm{N}(m_n(t),\sigma_n)[256\log_{10}(\cdot)], \end{aligned} \tag{2}$$

with the argument $[256\log_{10}(\cdot)]$ accounting for the exponentially increasing width of the 1024 fluorescence intensity-bins into which the flow cytometry data is discretized. Consequently, $\mathrm{M}_g(t)$ and $\mathrm{M}_n(t)$ are symmetric on a log-scale and skewed on a linear scale.

At $t = t_1$, the mean fluorescence intensities $m_g(t)$ and $m_n(t)$ are given by the parameters $m_{g,1}$ and $m_{n,1}$, respectively. At all times, the mean fluorescence intensities $m_g(t)$ and $m_n(t)$ have two contributions, (i) the natural background fluorescence of the cells, F_N, and (ii) the fluorescence conferred by the dye. The natural fluorescence remains constant, whereas the fluorescence conferred by the dye decreases over time with the cells' constant growth rates μ_g and μ_n, respectively, and with the rate of the unspecific dye loss, $\delta = 0.015\ \mathrm{h}^{-1}$ (cf. Supplementary Figure S2B). Hence, the

time progressions of the mean fluorescence intensities $m_g(t)$ and $m_n(t)$ within the observation window $[t_1, t_k]$ are given by

$$\begin{aligned} m_g(t) &= F_N + (m_{g,1} - F_N)\,\mathrm{e}^{-(\mu_g+\delta)(t-t_1)} \\ m_n(t) &= F_N + (m_{n,1} - F_N)\,\mathrm{e}^{-(\mu_n+\delta)(t-t_1)}\,. \end{aligned} \tag{3}$$

With the growth rate of the non-growing subpopulation empirically set to $\mu_n = 0.03\,\mathrm{h}^{-1}$, the optimal values $\mathbf{p_{opt}}$ of the remaining eight parameters

$$\mathbf{p} = [\mu_g \quad \sigma_g \quad \sigma_n \quad c_{g,1} \quad c_{n,1} \quad m_{g,1} \quad m_{n,1} \quad F_N] \tag{4}$$

are determined by cell count-weighted minimization of the sum-of-squares distance between the model (Equations 1, 2, and 3) and the data within the observation window $[t_1, t_k]$; hence, the optimal values $\mathbf{p_{opt}}$ for the eight parameters $\mathbf{p}$ are determined by

$$\mathbf{p_{opt}} = \min_{\mathbf{p}} \sum_{i=1}^{k} c(t_i)^{-1} \big(\mathrm{M}\,(t_i) - \Delta\,(t_i)\big)^{\mathrm{T}} \big(\mathrm{M}\,(t_i) - \Delta\,(t_i)\big). \tag{5}$$

The model fit in the observation window is used to calculate α, the fraction of cells adapting to growth on gluconeogenic substrates upon the substrate shift at t_0. This calculation is based on the definition of α,

$$\alpha = \frac{A_g(t_0)}{A(t_0)}, \tag{6}$$

with $A_g(t_0)$ the areal contribution of the $c_g(t_0)$ cells of growing phenotype, to $A(t_0)$, the total area of the total population's fluorescence intensity distribution at t_0, which comprises $c(t_0)$ cells in total. Because the area of any $\mathrm{N}\,(m, \sigma)$-distribution is unity, the cell count-scaled area of the distribution equals the number of cells; hence,

$$A(t_0) = c(t_0)\,. \tag{7}$$

Given $d(t)$ (see below), the number of cell divisions of the growing phenotype between t_0 and $t \in [t_1, t_k]$, the area $A_g(t_0)$ can be back-calculated from any area $A_g(t)$ within the observation window $[t_1, t_k]$ via

$$A_g(t_0) = \frac{A_g(t)}{2^{d(t)}}\,. \tag{8}$$

Within the observation window $[t_1, t_k]$, $A_g(t)$, the areal contribution of the growing subpopulation to the total population's fluorescence intensity distribution at time t, is given by

$$A_g(t) = c_{g,1}\, \mathrm{e}^{\mu_g (t - t_1)}\,. \tag{9}$$

The number of cell divisions of the growing phenotype between t_0 and $t \in [t_1, t_k]$, $d(t)$, is inferred from the loss of fluorescence intensity in this time period via

$$d(t) = \log\frac{1}{2} \log\frac{m_g(t_0) - F_N}{m_g(t) - F_N}\,, \tag{10}$$

with $m_g(t_0)$ the mean fluorescence intensity of the growing phenotype at t_0. At t_0, the mean fluorescence intensity of the growing subpopulation, $m_g(t_0)$, equals m_0, the mean fluorescence intensity of the total population; hence,

$$m_g(t_0) = m_0\,. \tag{11}$$

The mean fluorescence intensity of the total population at t_0, m_0, is determined through the minimization of the sum-of-squares distance between $\Delta(t_0)$ and the single Gaussian model

$$\mathrm{M}\,(t_0) = c(t_0) \cdot \mathrm{N}\,(m_0, \sigma_0)[256 \log_{10}(\cdot)]\,, \tag{12}$$

with σ_0 the standard deviation of the total population's fluorescence intensity distribution at t_0.

The beauty of this approach is that the determined values for α and μ_g are *independent* of the population's growth behavior between the shift to the gluconeogenic substrate at t_0 and the start of the observation window at t_1. This property is important because the quantification of the growth behavior within the early time period $[t_0, t_1]$ is highly uncertain due to, for instance, possible reductive cell divisions of the 'non-growing' phenotype and the early, gradual growth rate-acceleration of the 'growing' phenotype.

FBPase activity is inhibited by 2-deoxyglucose 6-phosphate

In the main text, we aimed to inhibit the enzyme Fbp. Fbp was reported to be inhibited by glucose 6-phosphate (Hines et al., 2007). However, if we would have added this compound to the medium, it could have been metabolized instead of the provided gluconeogenic carbon source. Instead, we thought to use the compound 2-deoxyglucose 6-phosphate, which is taken up by *E. coli* but not metabolized (Dietz and Heppel, 1971). However, we had to show that indeed this compound is able to inhibit the enzyme's catalytic activity.

Therefore, we overexpressed the His-tagged enzyme in *E. coli* using a plasmid, induced expression with 0.1 mM IPTG at 37 °C for 2 hours, purified it using a Ni^{2+} affinity column and determined its catalytic activity at 22 °C under different conditions (absence/presence of the known inhibitor glucose 6-phosphate, obtained from Sigma Aldrich; the putative inhibitor 2-deoxyglucose 6-phosphate, obtained from Santa Cruz Biotechnology). As buffer we used 50 mM HEPES (pH 7.5) with 20 mM $MgCl_2$. To monitor the reaction progression (i.e. depletion of fructose-1,6-bisphosphate, obtained from Sigma Aldrich), we developed an HPLC method as follows: Column: PL-SAX 1000Å 8 µm, 50 x 4.6 mm (40 °C) (Agilent); mobile phase: 0.1 M NaH_2PO_4 at pH 2.6; flow rate: 0.5 ml/min; detection: refraction index detector (35 °C); sample volume: 5 µl. Samples were automatically injected from the reaction vial every 7 min. The data from the different experiments, including controls, are shown in the figure below.

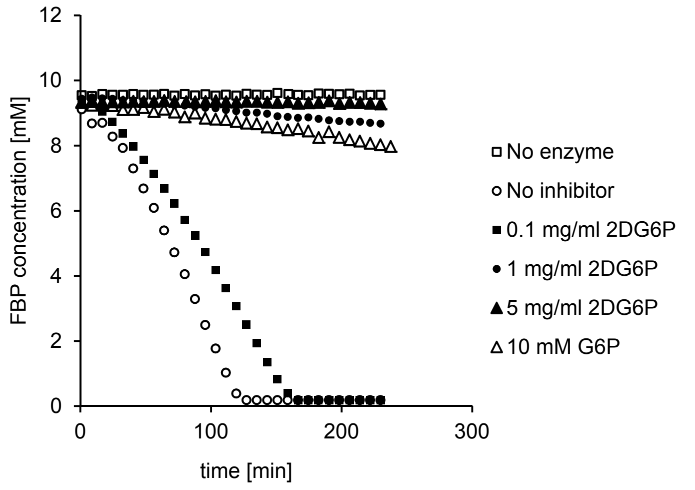

These results clearly indicate that also 2-deoxyglucose 6-phosphate is an inhibitor of Fbp activity and thus could be used in our experiment, which data is shown in Figure 4F.

Model of the bistability-generating molecular mechanism

In this section, we present the development and analysis of the mathematical model of the bistability-generating circuit shown in Fig. 5A of the main paper. We describe in detail the derivation of the model's differential and algebraic equations, and present the bifurcation analysis of the model underlying Fig. 5B and 6A. The aim of the model is to determine whether the mechanism identified in the main paper is capable to express the bistable phenotypes we observe experimentally.

In the main paper, we show that a line of reasoning based on published knowledge and experimental results leads to a candidate molecular mechanism. This candidate mechanism fulfills the pre-requisites for the generation of a bistable response: It involves cooperative binding events, a feedback architecture, and gene expression as a source of stochasticity. Here, we present the translation of this mechanism into differential equations and a bifurcation analysis of this system.

Note that the model of the candidate mechanism necessarily remains a simplification of much more complex cellular processes in *in vivo* central metabolism. However, if the candidate mechanism indeed captures the *dominant* regulatory process, then the neglected *in vivo* complexity only modulates but not fundamentally changes qualitative model behavior. To determine whether the candidate mechanism is indeed responsible for generating responsive diversification, the model is used to predict novel, qualitative perturbation effects. The experimental validation of these predictions, presented in the main paper, strongly suggests that the here modeled mechanism indeed operates *in vivo*.

<u>Summary of model development and analysis</u>

Here we present a brief summary of our model of the candidate regulatory mechanism (see Fig. 5A in the main text), and our model analysis. A more detailed outline of the model development follows in the next section.

To investigate if the model can generate the experimentally observed subpopulations, we described molecular interactions with mathematical equations, resulting in:

$$\frac{dE}{dt} = f(E, FBP_{SS}) = v_{e,max}\left(1 - \frac{FBP_{SS}^{n_e}}{FBP_{SS}^{n_e} + (K_{e,FBP})^{n_e}}\right) - d \cdot E \quad (13)$$

$$FBP_{SS}(J): \quad 0 = J - \frac{v_{Fbp,max}\frac{FBP_{SS}}{K_{Fbp,FBP}}\left(1+\frac{FBP_{SS}}{K_{Fbp,FBP}}\right)^3}{\left(1+\frac{FBP_{SS}}{K_{Fbp,FBP}}\right)^4 + L_{Fbp}\left(1+\frac{J \cdot K_{E_X,X}}{K_{Fbp,X}\cdot(v_{E_X,max} - J)}\right)^{-4}} \quad (14)$$

$$J(E, acetate) = \frac{k_{E,cat} \cdot E \cdot acetate}{acetate + K_{E,acetate}} \quad (15)$$

Equation 13 combines the regulation of E production through Cra activity and the regulation of Cra activity through FBP. $v_{e,max}$, $K_{e,FBP}$ and n_e parameterise the Hill-type kinetics of E production, and d represents the combined E degradation and dilution rate due to cell growth. Equation 14 yields the steady state concentration of FBP as an implicit function of the metabolic flux J (Supplementary Fig. S8A). $v_{E_X,max}$ and $K_{E_X,X}$ establish a flux-dependent concentration X, and $v_{Fbp,max}$, $K_{Fbp,FBP}$, $K_{Fbp,X}$ and L_{Fbp} parameterise the Monod-Wyman-Changeux kinetics of the tetrameric enzyme Fbp. Equation 15 is the Michaelis-Menten kinetics of E, parameterised by $k_{E,cat}$ and $K_{E,acetate}$, yielding J.

We found that this model structure tightly constrains the possible responses. This enabled us to focus on qualitative predictions without knowing the exact values of the poorly identifiable parameters. Some of the parameter sets representing biologically meaningful behavior (stable E dynamics, $E \geq 0$, $X_{SS} \geq 0$, $FBP_{SS} \geq 0$, $J \geq 0$) establish a bistable E concentration. Importantly, bistability in E is linked to bistability in J and growth. Through Equation 15, bistability in E propagates into bistability in J. As J fuels metabolism with essential biomass precursors, bistability in J further propagates into bistability in growth.

Model development

We here derive the differential and algebraic equations that model the architecture shown in Fig. 5A of the main text.

A 'super-enzyme' E subsumes the entire pathway from acetate uptake to fructose-1,6-bisphosphate (FBP) and is modeled with a Michaelis-Menten kinetic,

$$v_E(acetate, E) = \frac{k_{E,cat} \cdot E \cdot acetate}{acetate + K_{E,acetate}}, \tag{16}$$

where $acetate$ is the extracellular acetate concentration, $k_{E,cat}$ is E's maximal turnover rate, and $K_{E,acetate}$ is the Michaelis-Menten constant.

In *E. coli*, the production of most enzymes in this pathway is activated by the transcription factor Cra (Shimada et al., 2011b) FBP inactivates Cra by binding to it, forming Cra-FBP (Saier and Ramseier, 1996). The inhibition that FBP thus exerts on E production involves binding events -- FBP to Cra, and Cra to the promoter region of the e gene -- both of which may or may not involve cooperativity. The effect of FBP on e expression is lumped into the Hill-type function

$$f(FBP) = v_{e,max}\left(1 - \frac{FBP^{n_e}}{FBP^{n_e} + K_{e,FBP}^{n_e}}\right), \tag{17}$$

where $v_{e,max}$ is the maximal e expression rate, n_e is the degree of cooperativity, and $K_{e,FBP}$ is the FBP concentration required for 50% expression. For $n_e = 1$, the lumped binding processes do not contain cooperative binding events so that FBP exerts a parabolic influence on E production. For $n_e > 1$, these processes contain positively cooperative binding events, such that FBP exerts a sigmoidal influence on e expression.

With E degradation and dilution due to cell growth modelled as a linear function of E itself with slope d , the differential equation for E becomes

$$\frac{dE}{dt} = f(FBP) - d \cdot E. \tag{18}$$

The dilution of E due to cell growth depends on the growth rate μ, therefore d increases with μ. Also, the concentrations of ribosome and DNA polymerase complexes, which affect the rate of E synthesis, f, increase with μ. We assumed that these contributions cancel each other out and did not model the dependencies of d and f on μ. Although this simplification may introduce an error, it is still more desirable than the alternative, which would be the introduction of a high degree of error-prone structural uncertainty and complexity in an attempt to reduce the original error. Note that the dependency of f on ribosomes and DNA polymerases levels, and the dependencies of these levels on μ, are unclear. Further, μ itself would have to be modeled as a dependent variable. The structure of this function remains unclear, and previous attempts to identify it have failed even in much more detailed kinetic models of metabolism (Bettenbrock et al., 2006). Fbp represents the tetrameric enzyme fructose-1,6-bisphosphatase (Fbp), which converts FBP to fructose-6-phosphate. The enzyme Fbp is modelled with a Monod-Wyman-Changeux (MWC) kinetic with $n = 4$:

$$v_{Fbp}(X, FBP) = \frac{v_{Fbp,max} \dfrac{FBP}{K_{Fbp,FBP}} \left(1 + \dfrac{FBP}{K_{Fbp,FBP}}\right)^3}{\left(1 + \dfrac{FBP}{K_{Fbp,FBP}}\right)^4 + L_{Fbp}\left(1 + \dfrac{X}{K_{Fbp,X}}\right)^{-4}}, \tag{19}$$

where $v_{Fbp,max}$ is the maximal turnover rate, and L_{Fbp}, $K_{Fbp,X}$ and $K_{Fbp,FBP}$ are shape parameters of the MWC kinetic. Fbp is activated in a flux-dependent manner through the flux-signalling metabolite X, which is probably but not necessarily phosphoenolpyruvate or citrate (Hines et al., 2006). The flux-signalling metabolite X reports the flux J through E by strictly increasing with J. The metabolite X probably is, but may not be, located in the pathway subsumed into the super-enzyme E. If X is located within that pathway (as e.g. phosphoenolpyruvate or citrate), then J flows through X, and the level of X is determined by the metabolite's consuming enzyme kinetics (i.e. that enzyme

kinetic's 'flux resistance'). If the flux-signalling metabolite X is located outside the pathway subsumed into E (as e.g. adenosine-phosphate), then X signals J through an unknown, indirect mechanism. In either case, the important point is that the level of X strictly increases with the flux J in order to signal that flux. To achieve such a flux-dependent concentration of X, which is produced at rate J, the consumption of X follows the rate

$$v_{E_X}(X) = \frac{v_{E_X,max} \cdot X}{X + K_{E_X,X}}, \tag{20}$$

a Michaelis-Menten enzyme kinetics where $v_{E_X,max}$ is the maximal turnover rate, and $K_{E_X,X}$ is the Michaelis-Menten constant. The steady state concentration of the J-signalling metabolite X, X_{SS}, is then given by setting $v_{E_X}(X) = J$ and rearranging Equation 20 to

$$X_{SS}(J) = \frac{J \cdot K_{E_X,X}}{v_{E_X,max} - J}. \tag{21}$$

<u>Bifurcation analysis</u>

In this section, we perform a bifurcation analysis on the model equations derived in the previous section. This analysis results in Fig. 5B and 6A.

The steady state assumption on the fluxes

$$v_E = v_{E_X} = v_{Fbp} = J \tag{22}$$

allows the calculation of the steady state FBP concentration, FBP_{SS}, as function of J. Equations 19, 21 and 22 are combined to yield an implicit equation for FBP_{SS}:

$$FBP_{SS}(J):\ \ 0 = J - \frac{v_{Fbp,max}\dfrac{FBP_{SS}}{K_{Fbp,FBP}}\left(1+\dfrac{FBP_{SS}}{K_{Fbp,FBP}}\right)^{3}}{\left(1+\dfrac{FBP_{SS}}{K_{Fbp,FBP}}\right)^{4} + L_{Fbp}\left(1+\dfrac{J\cdot K_{E_X,X}}{K_{Fbp,X}\cdot(v_{E_X,max}-J)}\right)^{-4}}. \tag{23}$$

Through empirically probing the parameter space, we found that the model structure tightly constrains the possible responses. This enabled us to focus on qualitative predictions without knowing the exact values of the poorly identifiable parameters. By 'educated trial and error', we found parameter sets that lead to a stable E dynamics with $E > 0$, positive steady state metabolite concentrations ($FBP_{SS}(J), X_{SS}(J) > 0$), and an uptake of acetate ($J > 0$). These constraints resemble the system's biological function.

Of the parameter sets that lead to such biologically meaningful behaviour, we were specifically interested in those that enable two stable steady states, because a bistable acetate uptake rate can be interpreted as the experimentally observed 'growing' and 'non-growing' phenotypes. We found that such parameter sets exist, although it was harder to find these sets than it was to find sets that produce only one stable steady state. This suggests that the bistable region in parameter space is smaller compared to the monostable region.

However, the bistable region is not unrealistically small for the system to operate within this regime: First, we were able to find the bistable region within the 12-dimensional parameter space using only empiric means. Second, parameter values of a parameter set within the bistable region can be varied to a comfortable extent without leaving the bistable region. Third, parameter values that lead to bistable behaviour seem to be realistic. For instance, the parameters determining a substrate or effector concentration required for 50% activation are in the range of the respective compound concentrations. Further, we found that a high value of L_{Fbp} is essential to move the system to the bistable region. L_{Fbp} is a shape parameter of the MWC kinetics describing the tetrameric enzyme Fbp, which is substantially activated by X. Although a value for L_{Fbp} is not available in literature, another study (Bettenbrock et al., 2006) experimentally fitted the shape parameter L of the MWC kinetics

describing the tetrameric enzyme $PykF$, which is substantially activated by FBP, and obtained a similarly high value. This provides confidence that high values of L are realistic for tetrameric enzymes that are substantially activated by a metabolite. In the following, we report on our investigation of the system's operation in the bistable region. With parameters according to Table 0, Supplementary Fig. S8A shows the steady state metabolite concentrations (Equations 21 and 23) with varying steady state flux J. Note that X_{SS} monotonically increases with increasing J, whereas FBP_{SS} initially sharply increases, then gradually decreases, and finally increases again. The shape of the FBP_{SS} curve can be explained as follows. For small metabolic fluxes J, the X_{SS} concentration is too low to noticeably activate the enzyme Fbp. Therefore, Fbp can only provide the flux J if its substrate concentration FBP_{SS} is relatively high. When J is increased further, then the enzyme E_X can only provide a higher flux if it increases its substrate concentration X_{SS}. This increase in X_{SS} significantly activates Fbp so that this enzyme can provide the increased flux J with a relatively low substrate concentration FBP_{SS}. The activation effect of X_{SS} on Fbp however saturates with increasing X_{SS}. Therefore, if J and thereby X_{SS} are raised even further, then Fbp can only provide the flux J if the concentration of its substrate FBP_{SS} is increased again.

It is important to note that the thus established inverse dependence of FBP_{SS} on J within a wide range of J changes the sign of the closed feedback loop on E production from negative to positive.

Table: Parameters used in the simulations. [*]This notation means that the parameter was increased from 0.7 to 1.3, in steps of 0.1.

Parameter	Value in Suppl. Fig. 8A	Value in Suppl. Fig. 8B(C)	Value in Suppl. Fig. 8D(E)	Value in Suppl. Fig. 8B	Value in Suppl. Fig. 8C
$k_{E,cat}$	-	1	1	1	1
$K_{E,acetate}$	-	-	0.02 (0.1)	0.1	0.1
$v_{E_X,max}$	1	1	1	1	1
$K_{E_X,X}$	0.3	0.3	0.3	0.3	0.3
$v_{Fbp,max}$	1	1	1	1	1
$K_{Fbp,X}$	0.1	0.1	0.1	0.1	0.1
$K_{Fbp,FBP}$	0.1	0.1	0.1	0.1	0.1
L_{Fbp}	$4 \cdot 10^6$	$4 \cdot 10^6$	$4 \cdot 10^6$	$4 \cdot 10^6$	$4 \cdot 10^6$
d	-	0.18 (0.25)	0.18 (0.25)	0.35	0.35
$v_{e,max}$	-	1.1	1.1	1	0.7:0.1:1.3[*]
n_e	-	1 (2)	1 (2)	2	2
$K_{e,FBP}$	-	0.1 (0.45)	0.1 (0.45)	0.45	0.45

In a next step, we investigated how such a flux-dependent, S-shaped concentration profile of the metabolite FBP controls the expression of the enzyme E. With the parameters listed in the Table, Supplementary Fig. S8B reveals that the E production and degradation/dilution curves (the two terms in Equation 18) intersect at three points for both parabolic and sigmoidal regulation of E production. Of these three steady states, the middle one is unstable whereas the outer two are stable. Therefore, the system is capable of expressing bistable E concentrations.

The S-shaped form of the E production curve stems mainly from the S-shape of the FBP concentration curve (Supplementary Fig. S8A), which in turn is the result of cooperative allosteric activation of Fbp through X, as described previously. Hence, allosteric regulation of enzyme activity can be sufficient to generate bistability in gene expression if the allosterically introduced

effect of cooperativity on the metabolic level is propagated to the gene expression level via transcription factor-metabolite binding. Introduction of additional cooperativity in the process of propagating the FBP concentration to promoter activity pronounces the characteristic shape of the E production curve (Supplementary Fig. S8C), thereby making it 'easier' for the production and degradation/dilution curves to have three intersections. Thus, additional cooperativity widens the region of bistability.

Supplementary Fig. S8D and E show the steady state fluxes J through the pathway as a function of the $acetate$ concentration in the medium. Qualitatively, the same curve shapes are observed for both the parabolic and sigmoidal case, with a wider region of bistability for the latter. For low $acetate$ concentrations, only one stable steady state flux exists at a low level. When the $acetate$ concentration is increased, then, at a critical concentration, a second stable steady state flux at a high level is created along with an unstable steady state flux through a saddle-node bifurcation. Further increase of the $acetate$ concentration through the region of bistability can lead to a second saddle-node bifurcation where the unstable and the lower stable steady state fluxes collide and annihilate each other. For $acetate$ concentrations above this bifurcation point, only one stable steady state flux on a high level would remain. However, although extracellular $acetate$ concentrations can reach arbitrarily high levels, the capacity to take up $acetate$ is limited due to substrate saturation of the enzyme E. This saturation can cause the second saddle-node bifurcation to be unreachable, such that two stable steady state fluxes persist for arbitrarily high $acetate$ concentrations. Such behaviour resembles our experimentally observed bistable phenotypes, which we observed for arbitrarily high $acetate$ concentrations. These conditions lead to the bifurcation diagrams shown in Fig. 5B and 6A of the main text.

Omitted regulations support responsive diversification

As we have seen, the presented and analyzed core model, depicted in Fig. 5A of the main paper, is capable of reproducing the bistable phenotypes. However, the core model includes only a subset of all known regulatory interactions between the modeled components. In particular, the nuanced regulation

of Cra within the Embden-Meyerhoff-pathway has not been accounted for. This omitted nuanced regulation comprises (i) the repression of the production of several enzymes in lower glycolysis (Hardiman et al., 2010; Shimada et al., 2011a), and (ii) the activation of Fbp production (Chin et al., 1989; Ramseier, 1996; Shimada et al., 2011a), which catalyzes the gluconeogenic conversion of FBP to fructose-6-phosphate, and repression of PfkA production (Hardiman et al., 2010).

In the section below, we show that these omitted regulations in fact support the mechanism of responsive diversification that is established by those regulations that are included in the model. As we value a simpler model that includes only the core regulations responsible for generating responsive diversification more than a more complicated model that also accounts for these supporting additional interactions, we have not included these in the model.

1. Activation of Fbp and repression of PfkA production by Cra

Cra activates the production of the enzyme Fbp, which catalyzes the gluconeogenic conversion of FBP to fructose-6-phosphate, and represses the production of the enzyme PfkA, which catalyzes the reverse (glycolytic) reaction. These regulatory interactions are not included in the model.

Activation of Fbp production likely increases the abundance of the enzyme Fbp that consumes the metabolite FBP. A higher abundance of the enzyme Fbp means that a lower FBP concentration is necessary to achieve the same flux through Fbp (cf. our Fbp perturbation experiment shown in the main text). Hence, activation of Fbp production supports a low concentration of the metabolite FBP in gluconeogenic conditions. Similarly, Cra mediated repression of production of the glycolytic enzyme PfkA should lead to a lower abundance of PfkA in gluconeogenic conditions. As PfkA catalyzes the reverse glycolytic reaction (i.e. FBP formation from fructose-6-phosphate), a lower PfkA abundance discourages the glycolytic reaction and thus FBP production from fructose-6-phosphate is, which should lower the concentration of FBP.

In essence, both interactions support a low concentration of the metabolite FBP under gluconeogenic conditions, and therefore support the generation of responsive diversification established by the

modeled interactions.

2. Repression of enzyme production in lower glycolysis by Cra

The Embden-Meyerhoff pathway between PEP and FBP comprises a sequence of reversible reactions. Cra represses several of the enzymes catalyzing these reactions. This repression has not been included in the model.

A repression of the production of these *reversible* enzymes should lower their abundances. Yet, the lowered enzyme abundances are still sufficient to carry the gluoneogenic flux from PEP to FBP. The effect of these enzymes having lower abundances should be that the catalyzed reactions operate further away from their thermodynamic equilibrium and thus higher substrate concentrations are required to achieve the respective gluconeogenic flux. As PEP is the first substrate of this sequence of reversible reactions and FBP is its final product (the conversions between FBP and fructose-6-phosphate are achieved by two irreversible enzymes, see above), the concentration of PEP should rise. A high concentration of PEP activates the FBP-consuming enzyme Fbp and keeps FBP levels low; high PEP levels are essential for the modeled mechanism to generate two coexisting phenotypes. Therefore, the omitted repression of enzyme production in lower glycolysis by Cra further supports the generation of responsive diversification that is established by those core interactions that are included in the model.

Strains and plasmids used

Strains used.

Background	Mutation	Origin	Marker
BW25113	-	Keio collection	-
BW25113	Δ*acnA*	P1 from Keio collection	Kan
BW25113	Δ*acnB*	Keio collection	Kan
BW25113	Δ*pckA*	P1 from Keio collection	Kan
BW25113	Δ*ppsA*	P1 from Keio collection	Kan
BW25113	Δ*maeBsfcA*	P1 from Keio collection	-
BW25113	Δ*cra*	Keio collection	Kan
BW25113	Δ*fbp*	Keio collection	Kan
BW25113	Δ*dctA*	Keio collection	Kan
BW25113	Δ*iclR*	Keio collection	Kan
BW25113	Δ*crp*	Keio collection	Kan
BW25113	Δ*arcA*	Keio collection	Kan

Nucleotide sequences for primers used for verification of the mutant strains are provided as separate text file (FASTA).

Plasmids used.

Name	Backbone	Description	Origin	Marker
pP_{fbp}-gfp	pUA66	fbp-promoter, *gfpmut2*	this study	Kan
pP_{tac}-cra	pJF118HE	tac-promoter, *cra*	(Saka et al., 2005)	Amp
pP_{tac}-acnA	pJF118HE	tac-promoter, *acnA*	(Saka et al., 2005)	Amp
pP_{tac}-fbp	pJF118HE	tac-promoter, *fbp*	(Saka et al., 2005)	Amp
pP_{tac}-dctA	pJF118HE	tac-promoter, *dctA*	(Saka et al., 2005)	Amp

Supplementary References

Bettenbrock, K., Fischer, S., Kremling, A., Jahreis, K., Sauter, T., and Gilles, E.D. (2006). A quantitative approach to catabolite repression in Escherichia coli. J. Biol. Chem. *281,* 2578-2584.

Chin, A.M., Feldheim, D.A., and Saier, M.H.,Jr. (1989). Altered transcriptional patterns affecting several metabolic pathways in strains of Salmonella typhimurium which overexpress the fructose regulon. J. Bacteriol. *171,* 2424-2434.

Dietz, G.W., and Heppel, L.A. (1971). Studies on the uptake of hexose phosphates. I. 2-Deoxyglucose and 2-deoxyglucose 6-phosphate. J. Biol. Chem. *246,* 2881-2884.

Hardiman, T., Meinhold, H., Hofmann, J., Ewald, J.C., Siemann-Herzberg, M., and Reuss, M. (2010). Prediction of kinetic parameters from DNA-binding site sequences for modeling global transcription dynamics in Escherichia coli. Metab. Eng. *12,* 196-211.

Hines, J.K., Fromm, H.J., and Honzatko, R.B. (2006). Novel allosteric activation site in Escherichia coli fructose-1,6-bisphosphatase. J. Biol. Chem. *281,* 18386-18393.

Hines, J.K., Kruesel, C.E., Fromm, H.J., and Honzatko, R.B. (2007). Structure of inhibited fructose-1,6-bisphosphatase from Escherichia coli: distinct allosteric inhibition sites for AMP and glucose 6-phosphate and the characterization of a gluconeogenic switch. J. Biol. Chem. *282,* 24697-24706.

Ramseier, T.M. (1996). Cra and the control of carbon flux via metabolic pathways. Res. Microbiol. *147,* 489-493.

Saier, M.H.,Jr, and Ramseier, T.M. (1996). The catabolite repressor/activator (Cra) protein of enteric bacteria. J. Bacteriol. *178,* 3411-3417.

Saka, K., Tadenuma, M., Nakade, S., Tanaka, N., Sugawara, H., Nishikawa, K., Ichiyoshi, N., Kitagawa, M., Mori, H., Ogasawara, N., and Nishimura, A. (2005). A complete set of Escherichia coli open reading frames in mobile plasmids facilitating genetic studies. DNA Res. *12,* 63-68.

Shimada, T., Yamamoto, K., and Ishihama, A. (2011a). Novel members of the Cra regulon involved in carbon metabolism in *Escherichia coli*. J. Bacteriol. *193,* 649-659.

Shimada, T., Yamamoto, K., and Ishihama, A. (2011b). Novel members of the Cra regulon involved in carbon metabolism in Escherichia coli. J. Bacteriol. *193,* 649-659.
